# Rapid synthesis and decoration of reduced graphene oxide with gold nanoparticles by thermostable peptides for memory device and photothermal applications

**DOI:** 10.1038/s41598-017-10777-1

**Published:** 2017-09-08

**Authors:** Sachin V. Otari, Manoj Kumar, Muhammad Zahid Anwar, Nanasaheb D. Thorat, Sanjay K. S. Patel, Dongjin Lee, Jai Hyo Lee, Jung-Kul Lee, Yun Chan Kang, Liaoyuan Zhang

**Affiliations:** 1 Key Laboratory of Biopesticide and Chemical Biology, College of Life Sciences, Fujian Agriculture and Forestry University, Fuzhou, Fujian province 350002 PR China; 2 Department of Chemical Engineering, Konkuk University, Seoul, 05029 Republic of Korea; 3 Department of Mechanical Engineering, Konkuk University, Seoul, 05029 Republic of Korea; 4 Materials & Surface Science, Institute Bernal Institute, University of Limerick, Limerick, Ireland; 5 Department of Materials Science and Engineering, Korea University, Seoul, 02841 Republic of Korea

## Abstract

This article presents novel, rapid, and environmentally benign synthesis method for one-step reduction and decoration of graphene oxide with gold nanoparticles (NAuNPs) by using thermostable antimicrobial nisin peptides to form a gold-nanoparticles-reduced graphene oxide (NAu-rGO) nanocomposite. The formed composite material was characterized by UV/Vis spectroscopy, X-ray diffraction, Raman spectroscopy, X-ray photoelectron spectroscopy, field emission scanning electron microscopy, and high-resolution transmission electron microscopy (HR-TEM). HR-TEM analysis revealed the formation of spherical AuNPs of 5–30 nm in size on reduced graphene oxide (rGO) nanosheets. A non-volatile-memory device was prepared based on a solution-processed ZnO thin-film transistor fabricated by inserting the NAu-rGO nanocomposite in the gate dielectric stack as a charge trapping medium. The transfer characteristic of the ZnO thin-film transistor memory device showed large clockwise hysteresis behaviour because of charge carrier trapping in the NAu-rGO nanocomposite. Under positive and negative bias conditions, clear positive and negative threshold voltage shifts occurred, which were attributed to charge carrier trapping and de-trapping in the ZnO/NAu-rGO/SiO_2_ structure. Also, the photothermal effect of the NAu-rGO nanocomposites on MCF7 breast cancer cells caused inhibition of ~80% cells after irradiation with infrared light (0.5 W cm^−2^) for 5 min.

## Introduction

In last two decades, carbon materials such as graphite and carbon nanotubes have been studied extensively. Graphene is a 2-D carbon material with a honeycomb structure of conjugated sp^2^ carbon^1^. Because of its remarkable and unique physical, chemical, electrical, optical, and biological properties, graphene has gained attention in the scientific community and industry^2–4^. Graphene has been used to produce biosensors, biomolecules carrier, solar cells, catalysis agent, optoelectronics, batteries, solar cells (dye sensitized or organic) and for energy storage^5–8^. Most studies have focused on the chemical conversion of natural graphite for large-scale production of graphene or graphene oxide (GO).

To synthesize graphene, different physical, chemical, and biological methods have been developed^9^. Additionally, various strategies have been employed not only to isolate graphene sheets to form single sheets with or without changing its properties, but also to synthesize reduced form of GO^10–12^. Chemicals such as hydrohalic acid^13^, hydrazine^14^, *p*-phenylenediamine^15^, sodium borohydride^16^, and hydroquinone^17^ have been used to reduce GO. However, because of their explosive nature and toxicity towards biological materials, rGO is not used in biochemical applications^9^. Sonication was shown to exfoliate and reduce GO. However, because of the high sonication frequency required, structural damage may occur, which affects the physiochemical properties of the GO^18^. Thus, there is demand to produce reduced graphene using eco-friendly and rapid methods. For green reduction of GO, different biomolecules such as amino acids^19^, proteins (bovine serum albumin)^20^, microorganisms^21^, plant extracts^22^, sugars^23^, and ascorbic acid^24^ have been used. In the present study, thermostable peptides were used as bio-reductants to reduce GO at very high temperature in a very short time. To improve the electrical, thermal, and optical properties of GO, metal nanoparticles such as silver, copper, platinum, and gold (AuNPs) were decorated on the honeycomb structure^25^. Numerous studies have examined AuNPs-GO composites because of their wide range of applications in catalysis, sensors, diagnosis, and therapy, among others^26^. It is known that metal nanoparticle-decorated graphene oxides are promising materials for use in various optoelectronic applications because of their unique plasmonic properties^27^. This is essential to enhanced light-matter interaction in graphene for their use in optoelectronic devices. The incorporation of Au NPs in graphene may produce plasmonic effects that lead to the enhancement of optical absorption, resulting in improved performance of optoelectronic devices. Furthermore, the charge confinement in Au NP incorporation in graphene can create electrical bi-stability, demonstrating memory characteristics due to charge trapping and de-trapping^28^.

**Figure 1:**
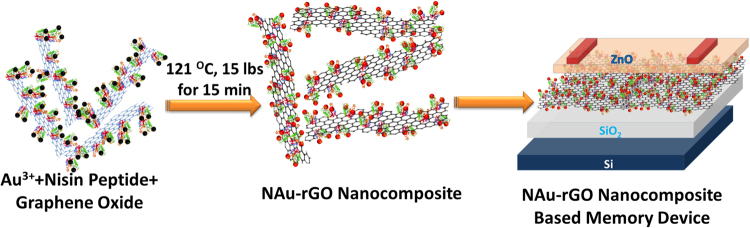
Schematics for the one-pot synthesis of NAu-rGO nanocomposite using thermostable nisin peptides and its application in TFT-based memory devices.

Different approaches have been used to form AuNPs-rGO composites. In the first approach, metal nanoparticles and GO are reduced separately and then mixed to form a composite, which is a time-consuming process. In another approach, metal nanoparticle precursors and GO are reduced in a single reaction. However, few studies have focused on the rapid biosynthesis of AuNPs-rGO composites in one pot. In this study, we demonstrate the rapid and simultaneous bio-reduction of GO and Au^3+^ to form rGO decorated with AuNPs at 121 °C for 15 min. Nisin is a lantibiotic that is a thermostable peptide, active at 121 °C for 15 min and stable at −20 °C^29^. It consists of 34 amino acids: ITSISLCTPGCKTGA- LMGCNMKTATCHCSIHVSK. *Lactococcus lactis* subspecies lactis isolated from milk-based products and vegetables contains nisin peptide. Nisin is an FDA-approved antimicrobial peptide used as a food preservative^30^. Nisin peptide has a wide range of antimicrobial activities against gram-positive microorganisms. Here, we present one-step synthesis of a gold nanoparticle–reduced graphene oxide (NAuNP–rGO) nanocomposite using thermostable antimicrobial nisin peptides in an autoclave. In a single pot, AuNPs and rGO were formed in a very short time. The reduced GO with dispersed AuNPs was obtained in a 15-min reaction. No toxic chemicals or harsh reaction conditions were used in the synthesis of NAuNP-rGO nanocomposites that would exclude it from being used for biomedical applications. Additionally, for electrochemical application, the NAu-rGO nanocomposite was used in a thin film transistor (TFT) for use in memory devices as shown in Fig. 1 where an enhanced response in transfer characteristics was observed. These formed NAu-rGO nanocomposites were evaluated for therapeutic application for NIR photothermal therapy (PTT), where enhanced photothermal activity of AuNPs because of the presence of rGO was achieved.

## Results and Discussion

### Characterization of rGO and NAu-rGO nanocomposite

**Figure 2:**
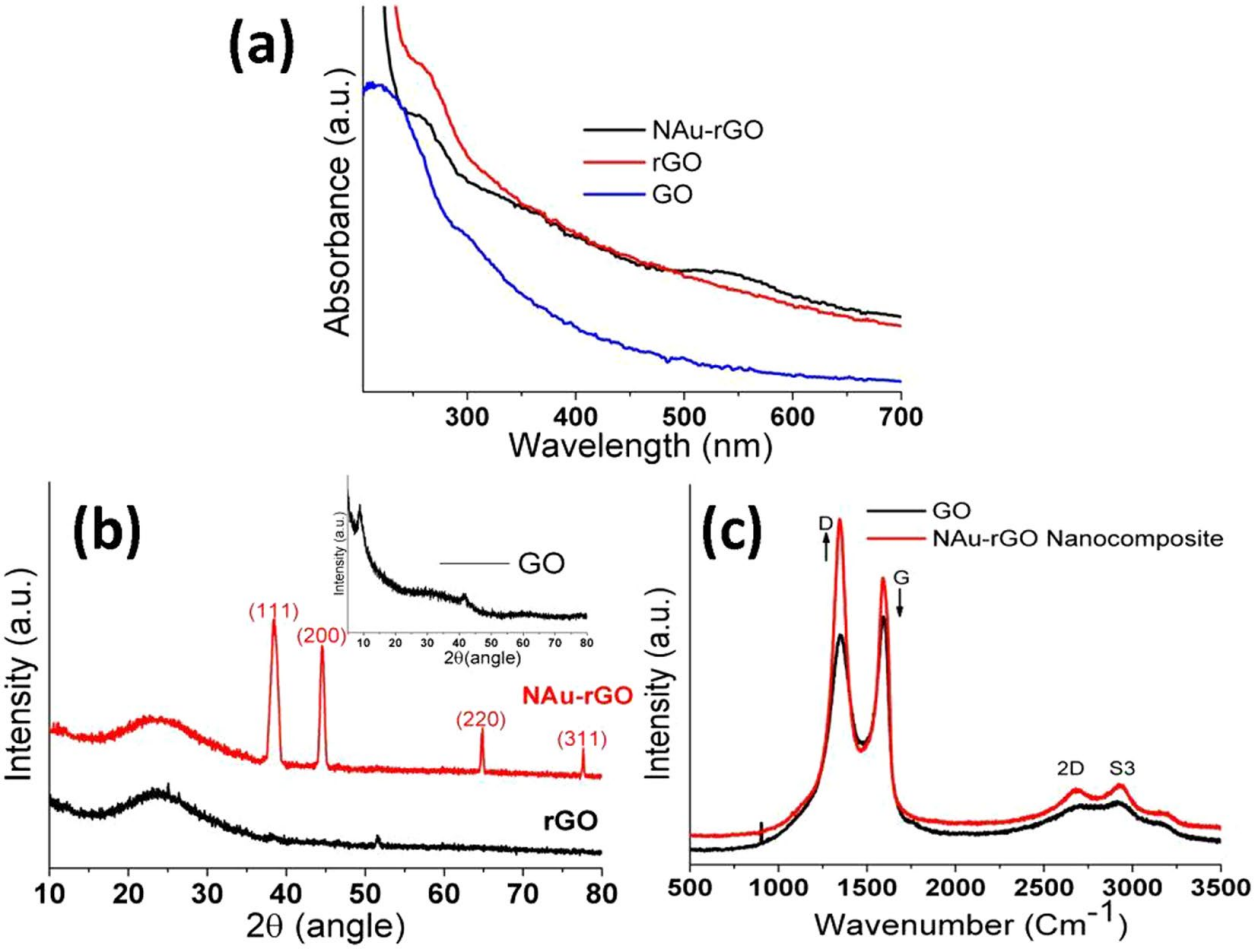
Characterization of the GO, rGO, and NAu-rGO nanocomposite. (**a**) UV–Vis spectra of GO (blue), rGO (red), and NAu-rGO nanocomposite (black). (**b**) XRD patterns of rGO (black), NAu-rGO(red) (Inset: XRD patterns of GO) (**c**) Raman spectra of GO (black) and NAu-rGO nanocomposite (red).

The reduction of GO and formation of NAu-rGO nanocomposite was initially analyzed by UV-Vis spectrometry. Figure 2a shows the UV-Vis absorption spectra of GO, reduced GO (rGO), and NAu-rGO nanocomposite. GO showed exhibited an absorption maximum at 230 nm because of the π → π* transition of the involving C = C bonds (Fig. 2a; blue). UV-Vis spectroscopy also showed absorption at ~300 nm in GO because of the n → π* transition of the C = O bonds (Fig. 2a; blue). The red shifting in the absorbance towards ~270 nm was observed after reduction the reducing reaction, indicating formation of rGO (Fig. 2a; red), which agrees with observations of previous reports^31^. Simultaneous reduction of GO and Au^3+^ formed the NAu-rGO nanocomposite. The appearance of an absorption peak at ~270 nm for rGO and ~530 nm for NAuNPs was observed by UV-Vis spectroscopy (Fig. 2a; black), clearly indicating the formation of NAuNPs on the surface of reduced graphene sheets.

The crystal structure of GO, rGO, and NAu-rGO nanocomposite was analyzed by XRD, which was also used to determine the interlayer distances of GO and rGO (Fig. 2b). The 2θ diffraction peak at 10.30° of GO showed a relative interlayer distance of 0.85 nm, indicating the presence of oxidized graphite layers. After reduction by thermostable peptides, rGO showed a broad diffraction peak at 25.6°, suggesting that GO was successfully reduced^32^. The XRD pattern of the NAu-rGO nanocomposite showed peaks at 2θ values of approximately 38.20°, 44.48°, 64.58°, and 77.60°, which were assigned to the (111), (200), (220), and (311) crystallographic planes of cubic structure of elemental NAuNPs, respectively (Fig. 2b), confirming the formation of NAuNPs in the composite. The XRD pattern is matched with JCPDS card No 004–0784.

For more information related to structural and electrical properties, Raman spectroscopy of the rGO and NAu-rGO nanocomposite was performed. Figure 2c shows the Raman spectra of the GO powder (black) and NAu-rGO nanocomposite (red). In graphene, the G peak and 2D peak represent sp^2^-hybridized carbon-carbon bonds. The G peak showed a band at ~1593 cm^−1^ and that of D was at ~1345 cm^−1^, corresponding to the A_1g_ mode and E_2g_ mode of the carbon atom, respectively. A change in the electronic conjugation state is indicated by a change in the I_D_/I_G_ ratio. The I_D_/I_G_ ratio before reduction was 0.9 for GO, and 1.22 for the NAu-rGO nanocomposite, demonstrating that at the end of the process, the sp^2^ domain was reduced in size^33^. The graphene sheets possibly showed a break during reduction, creating greater numbers of sp^2^ domains, which indicated that the reduction process had occurred^34^. Additionally, in the NAu-rGO nanocomposite, increased intensities of the 2D peak (~2681 cm^−1^) and S3 peak (~2931 cm^−1^) indicated improved graphitization and the presence of few layered graphene sheets in the NAu-rGO nanocomposite. Additionally, in the NAu-rGO nanocomposite, increased intensities of the 2D peak (~2681 cm^−1^) and S3 peak (~2931 cm^−1^) indicated improved graphitization and the presence of few layered graphene sheets in the NAu-rGO nanocomposite^35^.

**Figure 3:**
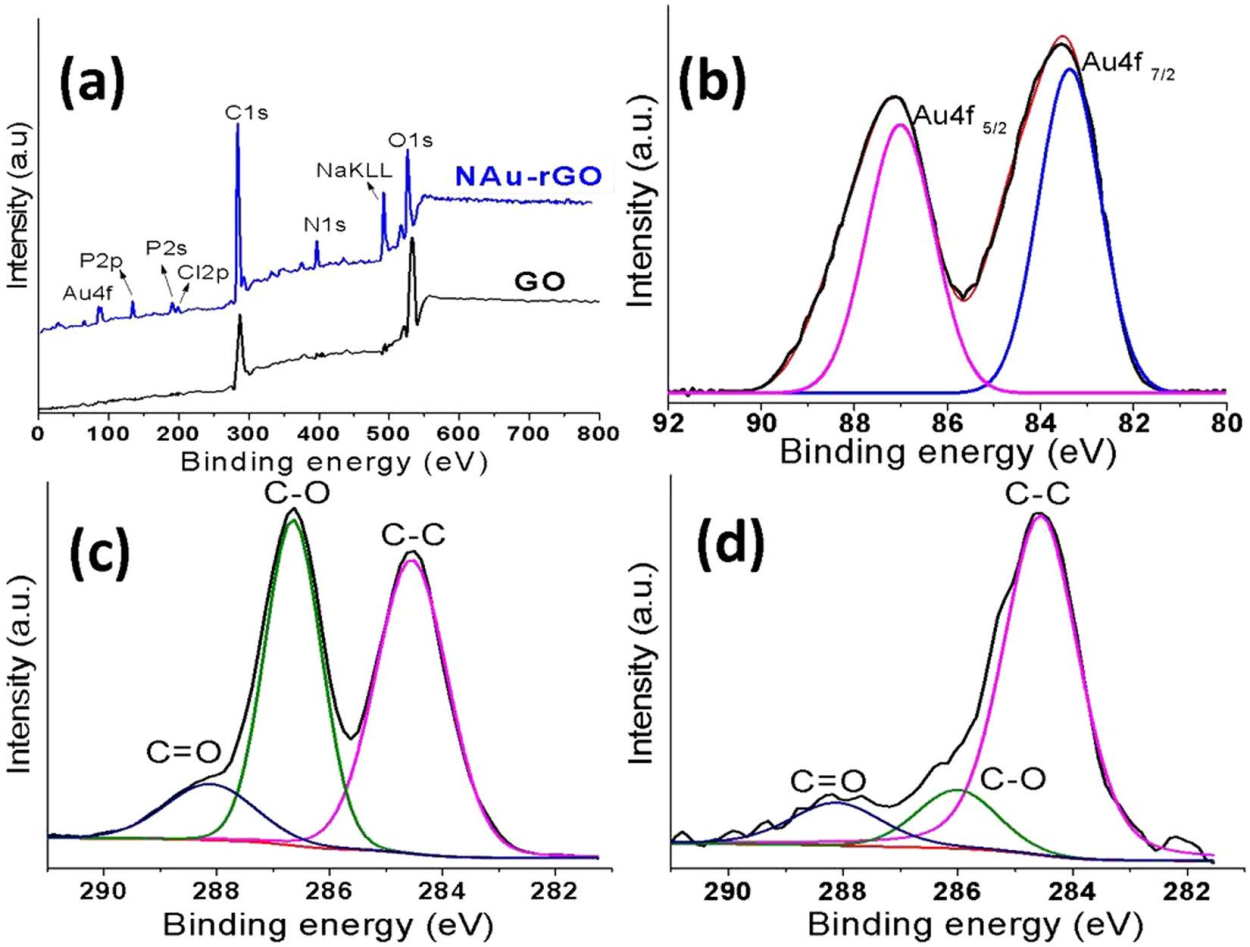
High resolution XPS Spectra of GO, rGO, and NAu-rGO nanocomposite. (**a**) XPS survey scan of GO and NAu-rGO nanocomposite. (**b**) High-resolution XPS spectrum of Au 4 f pattern for NAu–rGO nanocomposite. High-resolution spectra of C 1 s of (**c**) GO and (**d**) NAu-rGO nanocomposite.

Figure 3a shows the XPS survey spectra of the GO and NAu-rGO nanocomposite, which shows characteristic peaks for C1s and O1s. The survey spectra also revealed the presence of Au 4 f and N 1 s elements in the NAu-rGO nanocomposite. From the survey spectra, O1s peak was clearly weakened in the NAu-rGO nanocomposite as compared to GO and the peak associated with C1s was predominant in NAu-rGO nanocomposite compared to in GO. An Au 4 f peak was observed by XPS of the NAu-rGO nanocomposite after reduction of Au^3+^ to Au°. Additionally, the presence of N 1 s in the NAu-rGO nanocomposite indicate peptides on the surface of the rGO. Figure 3b shows characteristic XPS spectra of metallic Au°, which is centred at 83.6 and 86.9 eV, suggesting the formation of AuNPs on the surface of NAu-rGO nanocomposite^36^. The contribution of carbon-oxygen binding arrangement in GO and NAu-rGO nanocomposite was demonstrated by high-resolution XPS spectra of region C 1 s (Fig. 3c and d). Two main components were present from C=C/C–C at ~284.56 eV and C–O at ~286.61 eV, and one minor component from C=O at 288.1 eV in high-resolution spectra from C1s of GO (Fig. 3c)^37^. After reduction by thermostable nisin peptide, the intensities of the oxygen-dominated groups (hydroxyl and epoxy groups) were remarkably decreased and the C–C bond remained dominant as a single peak at higher energy (Fig. 3d). These results confirm that the structure of the thermostable peptide significantly changed the structure of GO during the reduction process^19^.

**Figure 4:**
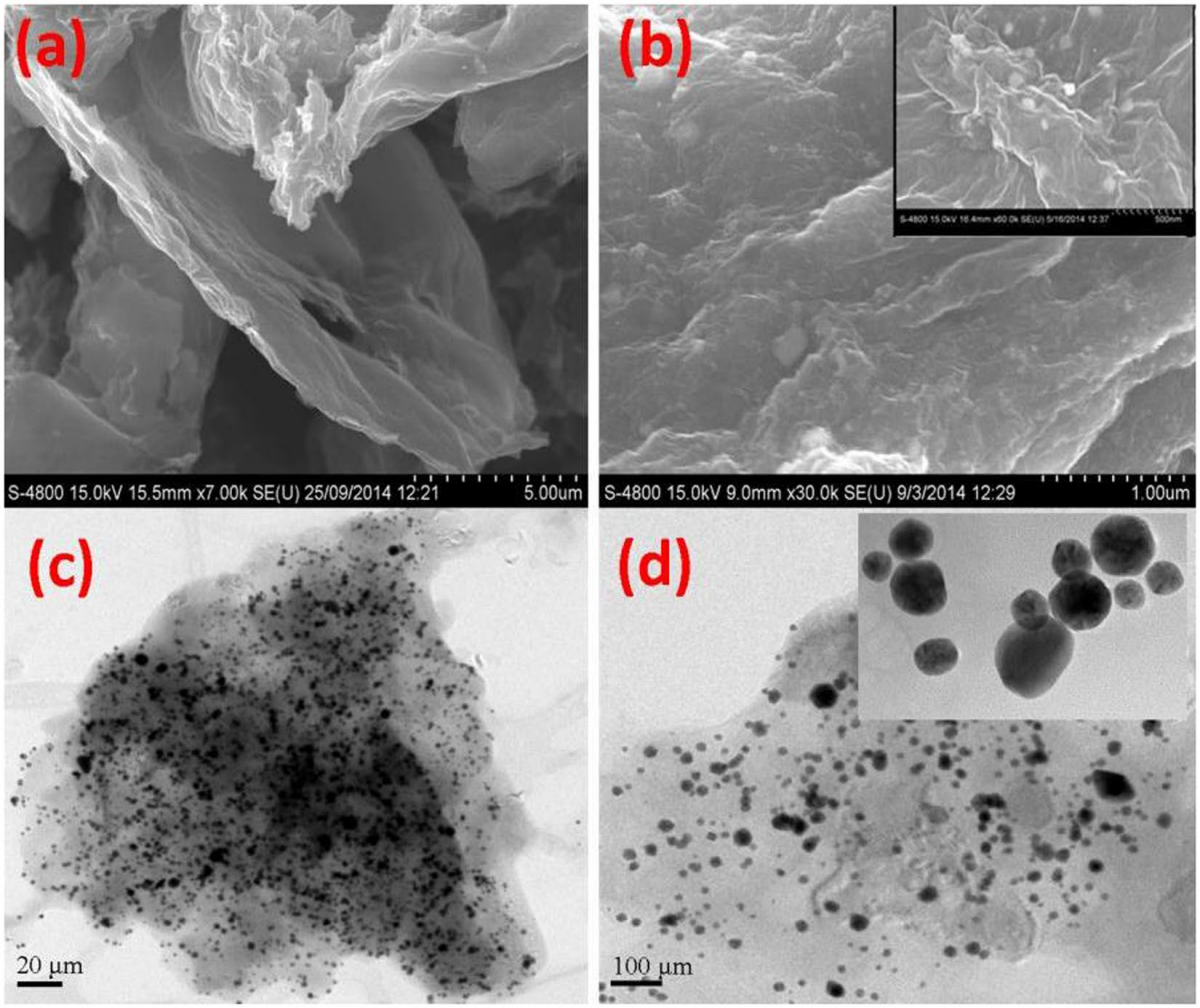
Electron microscopy studies of rGO and NAu-rGO nanocomposite. FE-SEM micrograph of (**a**) rGO and (**b**) NAu-rGO nanocomposite. Inset: right-top corner of (**b**) showing magnified FE-SEM image of NAuNPs formed in the rGO. High-resolution TEM (HR-TEM) micrograph of (**c**) and (**d**) NAu-rGO nanocomposite. Inset: right-top corner of (**d**) showing HR-TEM image of NAuNPs formed in the graphene sheets.

Figure 4a and b shows FE-SEM micrograph of the rGO and NAu-rGO nanocomposite. The clear surface of the rGO is shown in Fig. 4a, whereas the NAu-rGO nanocomposite showed a rough surface in Fig. 4b because of the presence of AuNPs. The presence of NAuNPs on the surface of rGO was evident in magnified FE-SEM micrograph (Fig. 4b; Inset). Figure 4c,d show HR-TEM micrographs of the as-prepared NAu-rGO nanocomposite. The AuNPs were dispersed on the surface of rGO and nearly unaggregated, but in a polydispersed form (Fig. 4d; Inset). All AuNPs were present on the surface of the rGO and did not protrude from the surface, indicating very strong interactions between rGO and NAuNPs through nisin peptides. The average diameter of the AuNPs was 25 nm, which agrees with the size obtained by XRD analysis. The Energy-dispersive X-ray spectroscopy (EDS) study confirmed that the formed nanoparticles on the rGO surface are of Au NPs (Supplementary Fig. 1) showing the characteristics signals of elemental gold metal^36^.

Different synthetic and biological chemicals were used to reduce GO (Table 1), such as L-cysteine, bovine serum albumin, and hormone, under very high alkaline conditions. Microorganisms, plant extracts, and some amino acids reduce GO at room temperature or high temperature under alkaline conditions, but the process is very time consuming. Thus, we used thermostable antimicrobial nisin peptides to reduce GO under neutral condition. Various chemicals could simultaneously reduce the AuNPs and GO to form AuNPs-rGO nanocomposites. Table 2 shows the different chemicals and reaction conditions used to synthesise AuNPs-rGO nanocomposites. Some reducing components required high alkaline and high temperature conditions for the reaction. For tyrosine-, bovine serum albumin-, ascorbic acid-, and tannic acid-mediated synthesis of AuNPs-rGO nanocomposites, rGO was first synthesized and then used as a template for AuNPs synthesis. In this study, the thermostable nisin peptide was used for one-step reduction, which is an efficient and rapid reduction process for forming AuNPs-rGO nanocomposites.

**Table 1:** Comparison of graphene reduction by different biological reducing agents.

Bio-reducing Material	Reduction Condition	I_D_/I_G_ ratio	Doping	Reference
**Amino Acid**				
L-Glutathione	50 °C, 6 h	—	—	39
L-Cysteine	NaOH, RT, 72 h	1.17	Adsorbed	37
Glycine	95 °C, 36 h	1.09	N-doped	41
L-Lysine	95 °C, 9 h	1.11	N-doped	42
**Microorganisms**				
*Shewanella*	Anaerobic, 72 h/Aerobic, 60 h	—	Adsorbed	41, 44
*E. coli*	37 °C, 48 h	0.97	—	21
**Plant Extracts**				
Tea polyphenols	90 °C, 2.5 h	—	Adsorbed	45
*R. damascena*	95 °C, 5.5 h	1.09	Adsorbed	46
β carotene	95 °C, 24 h.	1.01	—	47
**Proteins**				
Bovine serum albumin	NaOH, 55–90 °C, 3–24 h	—	Adsorbed	20
Hormone (Melatonin)	NH_3_, 80 °C, 3 h	—	Adsorbed	48
**Thermostable Peptide** (Nisin)	121 °C, 15 min	1.22	Adsorbed	This work

**Table 2:** Comparison table for the reduction of gold-graphene by different chemical reducing agents.

Reducing Material	Reduction Condition	Number Steps	Reference
Ascorbic Acid, NaBH_4_	80 °C, 6 h	Two	24
*Azadirecta indica*	25 °C, 24 h	One	50
Rose water	95 °C, 5 h	One	46
DNA	NaOH, 95 °C, 5 h	Two	51
Diastase	NH_4_OH, 90 °C, 6 h	One	49
Bovine serum albumin	NaOH, 55–90 °C, 3–24 h	Two	20
Tyrosine	100 °C, 3 h	Two	52
Thermostable Peptide (Nisin)	121 °C, 15 min	One	This work

The mechanism of the reduction process is not still clear. Due to the thiol groups of peptides, high temperature, and high pressure, the formation of AuNPs-rGO nanocomposites may have occurred. The thermostable nisin peptide consists of thiol group containing amino acids like methionine and cysteine, which may act as hydrogen donor to the oxygen moieties of graphene oxide^37^. The carbonyl, hydroxyl, and epoxide groups contribute for the oxygen moiety in graphene oxide^38^. Under mild basic condition, the oxygen containing carbonyl and epoxide groups are converted to hydroxyl group and thiol group containing amino acids reacts with hydroxyl group with removal of water molecule. Diez *et al*. demonstrated reduction of graphene oxide under high pressure of 80 bar at 180 °C, whereas in this present work, 1.034 bar (15 lbs) at 120 °C was used for the reduction process^39^. Thus, there was very negligible role of pressure and temperature for the reduction reaction. In our previous work, we have demonstrated the synthesis of gold nanoparticles using thermostable antimicrobial nisin peptides at autoclave reaction condition^40^. The antimicrobial peptide remained active even after formation of AuNPs at 121 °C. Au^3+^ ions have affinity towards thiol groups forming Au – S bonds, which converts Au^3+^ to Au° under high temperature. Therefore, the thiol group contributed for the simultaneous and rapid reduction reaction to form reduced graphene oxide and NAuNPs under mild pressure and high temperature. These nisin peptides also contribute to stabilization of the AuNPs as well as rGO.

### NAu-rGO nanocomposites non-volatile TFT memory characteristics

The use of rGO is widely focused on optoelectronic applications because of its superior electrical and optical properties. The Fermi levels and work function of graphene and rGO can be tailored via chemical doping. This property has been utilized recently in device applications such as high-efficiency chemically doped solar cells. Therefore, rGO is expected to provide a multi-band structure to effectively manipulate charge carrier trapping behaviour in TFT memory devices. Several research groups have tested GO and rGO as charge-trapping media for organic TFT non-volatile memory devices and obtained satisfactory results^53, 54^. A simple approach for modulating the trapping behaviour of solution-processed ZnO TFT non-volatile memory devices involves placing NAu-rGO nanocomposites in dielectric layers.

**Figure 5:**
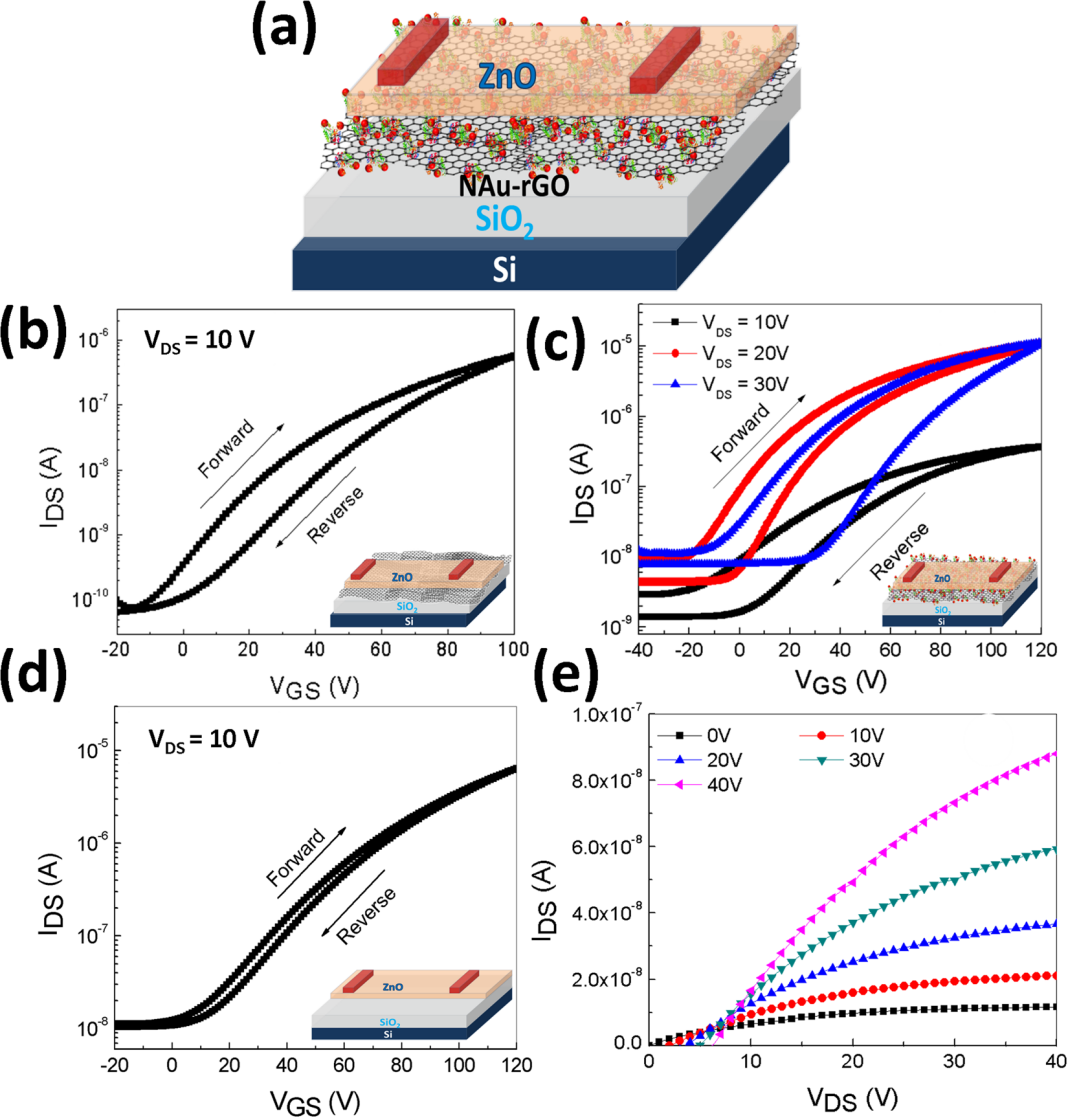
Construction and characteristics of NAu-rGO based TFT. (**a**) Schematic of the ZnO TFT memory device. (**b**) Transfer characteristics of the ZnO TFT memory device embedded with rGO at ZnO and SiO_2_ interface. (**c**) Transfer characteristics of the ZnO TFT memory device embedded with NAu-rGO nanocomposite at ZnO and SiO_2_ interface with varying drain voltage (V_DS_). (**d**) Transfer characteristics of ZnO TFT embedded without NAu-rGO nanocomposite. (**e**) Output characteristics of ZnO TFT embedded with NAu-rGO nanocomposite at ZnO and SiO_2_ interface.

A schematic of the NAu-rGO nanocomposite-based ZnO TFT memory device is presented in Fig. 5a, on which a layer of NAu-rGO nanocomposite was introduced in-between the SiO_2_ and ZnO layers. Transfer characteristic [Drain current (I_DS_) vs. gate voltage (V_GS_)] of ZnO TFT memory device embedded with rGO at ZnO and SiO_2_ interface is shown in Fig. 5b. The transfer curve exhibits small hysteresis window during the double sweep mode. Figure 5c shows the transfer characteristics of the ZnO TFT embedded with NAu-rGO nanocomposite in-between ZnO semiconductor and SiO_2_ dielectric layers with various drain voltage (V_DS_). A clear clockwise wider hysteresis I_DS_-V_GS_ curve was obtained by sweeping V_GS_ in the forward (−40 V to 120 V) to reverse (120 V to −40 V) voltage direction. It is seen from the figure that the obtained hysteresis became wider with varying drain voltage from 10 to 30 V, indicating more charge carriers are trapped at the charge trapping media in-between ZnO semiconductor and SiO_2_ dielectric layer. This suggests that the large hysteresis is attributed to electron/hole trapping in the NAu-rGO nanocomposite during the forward and reverse voltage sweep. Comparison of the reference device with the no NAu-rGO nanocomposite (Fig. 5d) revealed a low hysteresis characteristic, confirming that the presented memory TFT operated through electrostatic charging and discharging of the NAu-rGO nanocomposite rather than the interface between ZnO and SiO_2_ dielectric layers. Large numbers of structural defects were formed in the chemically synthesised NAu-rGO nanocomposite, which were the main cause of charge trapping of the NAu-rGO nanocomposite ZnO TFT memory device. However, the ratio of sp^2^ and sp^3^ fractions can be tuned by additionally reducing GO. Therefore, its band gap can be easily altered and then controllability transforms GO from an insulator to a semiconductor and to a graphene-like semi-metal. It is thought that the semi-metallic nature of rGO, having a covalently bonded honeycomb lattice structure, overcomes various defects including holes, which play an important role as charge trapping sites in charge trap memory devices. Transfer characteristics of ZnO TFT embedded with NAu-rGO nanocomposite exhibited a wider hysteresis window than that observed for various Au NP- and r-GO-based nano-floating gate dielectrics^55, 56^. Figure 5e shows the output characteristics of the ZnO semiconductor layer with SiO_2_/NAu-rGO nanocomposite dielectric stack memory TFT as the gate voltage was varied from 0 to 40 V. The drain current increased with increasing gate voltage and reached saturation because of pinching-off of the active channel of the transistor, exhibiting typical n-type transistor behaviour. The electrical parameters of a ZnO TFT non-volatile memory device embedded with NAu-rGO nanocomposites at the interface of ZnO and SiO_2_ were calculated by applying standard MOSFET equations to its transfer characteristic. The saturated mobility was extracted to be 0.52 cm^2^/V.s. Forward and reverse threshold voltage was observed to be 14.52 and 45.52 V, respectively. The on/off current ratio was found to be in the range of 10^3^.

**Figure 6:**
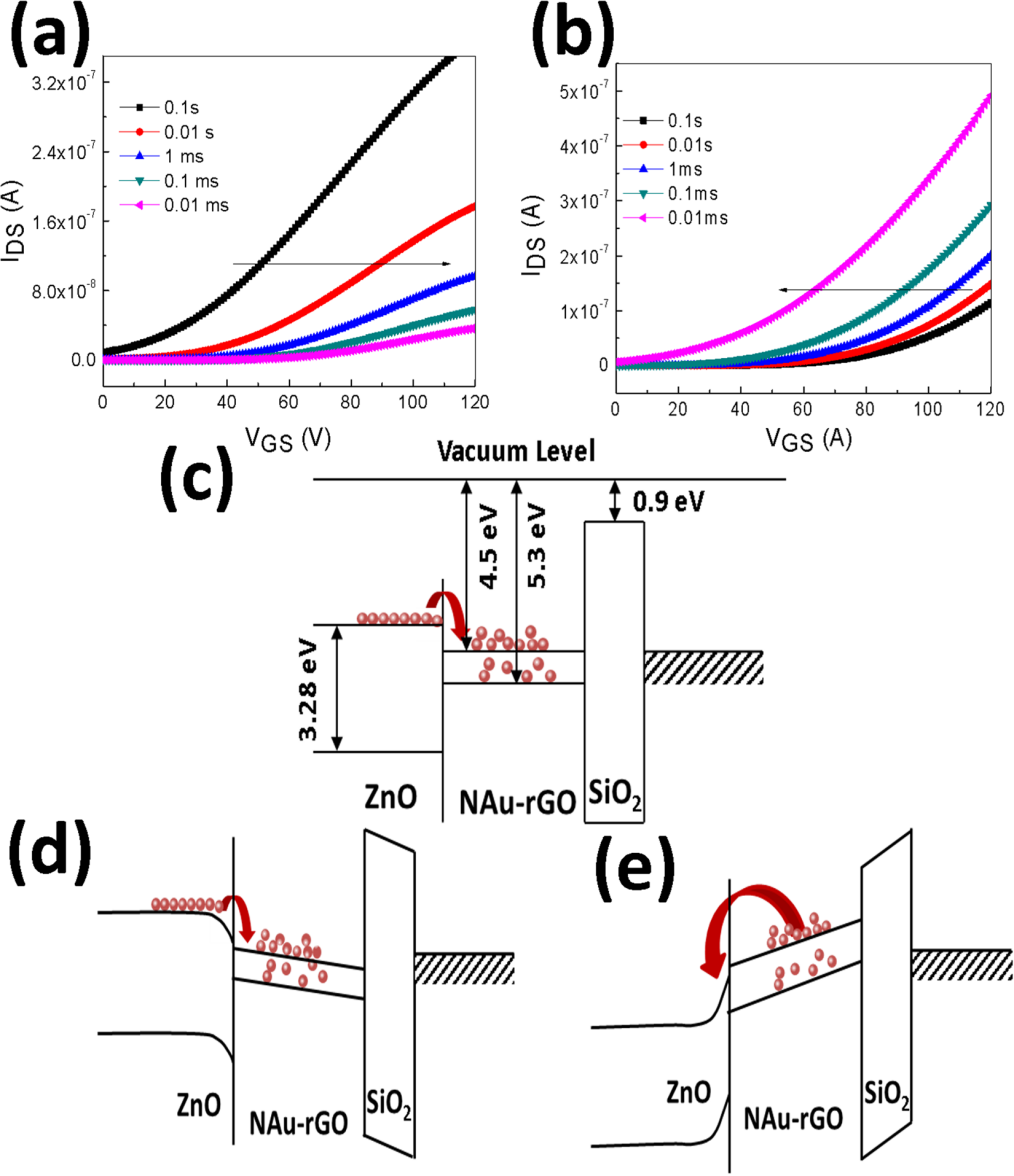
Transfer characteristics of ZnO-TFT embedded with NAu-rGO nanocomposite with respect to programming time. (**a**) Writing characteristics and (**b**) erasing characteristics. (**c**) Schematic diagram band gap diagram of ZnO-TFT-based non-volatile memory device embedded with NAu-rGO nanocomposite without applying electric field. (**d**) Programming state. (**e**) Erasing state.

To identify the switching behaviour of the NAu-rGO nanocomposite-based ZnO memory TFT, a gate bias of ±50 V was applied for the writing and erasing processes. Under the positive voltage bias condition (writing process), a positive voltage threshold voltage shift (∆V_th_) was observed in the transfer characteristics. Figure 6a shows the increased positive shift of transfer curves with increasing programming time. After programming at +50 V for 0.1 s, ∆V_th_ increased from 22.25 V for a programming time of 0.1 s to 61.30 V for a programming time of 0.01 ms. Figure 6b shows the transfer curves of erasing characteristics. After programming at −50 V for 1 s, a negative erasing gate pulse was applied for an erasing time of 1 s as the source, drain, and channel regions were grounded. Upon applying a −50 V gate bias (erasing process), the transfer curve shifted down by approximately 40–50 V. These reversible threshold voltage shift behaviours of NAu-rGO nanocomposite-based ZnO memory TFT may be related to carrier trapping and de-trapping in the NAu-rGO nanocomposite, suggesting that the NAu-rGO nanocomposite can be used as a charge trap layers for memory device application.

Figure 6c shows a simple description of charge trap and de-trap processes in the form of schematic band diagram of a ZnO-TFT-based non-volatile memory device embedded with NAu-rGO nanocomposite at the interface of ZnO semiconductor and SiO_2_ dielectric layers. After applying positive voltage during the programming stage, electrons accumulated in the channel were transferred into the NAu-rGO nanocomposites. It is expected that most electrons were trapped at the NAu-rGO nanocomposites (Fig. 6d). These trapped electrons occupied through shallow level states to deep level states. Charge trapping energy levels in chemically synthesized NAu-rGO nanocomposites may be attributed to structural defects and the work functions of NAu- rGO, the channel layer, and the gate dielectric. Large numbers of structural defects form in the chemically synthesized NAu-rGO nanocomposites and are a primary cause of charge trapping in related devices. Additionally, there were several oxygen groups between the rGO and NAu-rGO nanocomposites. These oxygen groups trapped charge carriers efficiently. Therefore, additional trapping levels were present with the NAu-rGO nanocomposites. During the erasing process, electrons occupied at a shallow level can be firstly de-trapped and transported to the channel, and then deep level state-trapped electrons were de-trapped (Fig. 6e). This study is particularly innovative because it uses a nanocomposite with a core–shell structure that functions a charge storage node.

### Photothermal activity of the nanocomposite

**Figure 7:**
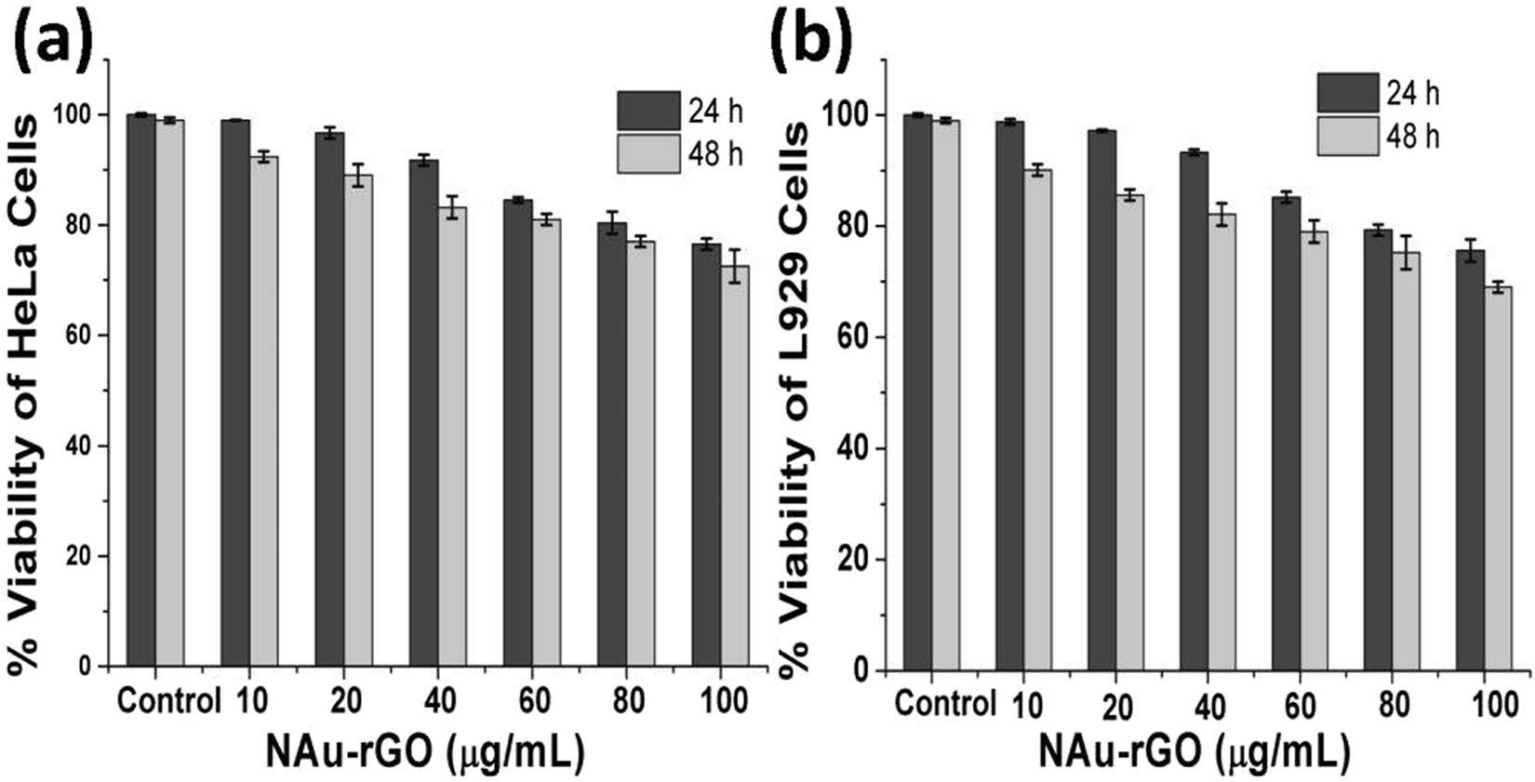
Cytotoxicity studies of NAu-rGO nanocomposite. (**a**) HeLa cell line, (**b**) L929 cell lines for 24 and 48 h using MTT assay.

Minimally invasive treatment methods such as near infrared PTT have gained attention over the past few decades. Various biomolecule-functionalized rGOs and Au-functionalized rGOs have been used as photothermal active nano-formulations against cancerous cells using NIR-wavelength laser illumination, and photothermal conversion efficacy and biocompatibility have been examined^57–59^. Here, to demonstrate the biomedical application of the peptide-synthesized NAu-rGO nanocomposite *in vitro*, PTT was performed on breast cancer cells (MCF7 cells), where cells were irradiated with NIR radiation using an 800-nm diode laser (power = 0.5 W/cm^2^) for varied times with GO, NAuNPs, and NAu-rGO nanocomposites. Here, to demonstrate the biochemical application of the biological synthesized NAu-rGO nanocomposite, *in vitro* PTT was performed on breast cancer cells (MCF7 cells), where cells were irradiated with near infra-red radiation using an 800-nm diode laser (power = 0.5 W/cm^2^) for varied times with GO, NAuNPs, and NAu-rGO nanocomposite. Cell viability was tested for 2 and 24 h after each exposure time. For biomedical application of the NAu-rGO nanocomposite, cytotoxicity analysis was performed on cervical cancer cells (HeLa cell line) and fibroblast cells (L929 cell line) using the MTT assay, where cells were grown in presence of NAu-rGO nanocomposite for 24 and 48 h (Fig. 7). In both cells, dose-dependent cytotoxicity was observed. Up to 40 μg/mL concentration, both cells showed ~90% viability for 24 and 48 h incubation. A further increase in the concentration showed increased toxicity towards both cell lines. Therefore, 10 μg/mL of NAu-rGO nanocomposite was used and compared with the same concentration of GO and NAuNPs as controls. UV-Vis-NIR spectroscopy analysis was performed for GO, rGO, and NAu-rGO nanocomposite to examine their absorbance in the NIR region before application of the as-prepared nanocomposite for photothermal activity (Supplementary Fig. 2). The NAu-rGO nanocomposite showed absorbance at ~540 nm, which is the characteristic absorbance of Au NPs, but showed no absorbance at 800 nm. From TEM micrographs (Fig. 4c and d), it is evident that the formed NAu NPs on rGO are varied in size, which may be the reason for the absorbance in visible and NIR regions. A similar phenomenon was observed by Komarala *et al*. and Sharma *et al*. where gold nanospheres decorated with layered double hydroxides (LDHs) and silica nanoparticles showed no absorbance in the NIR region, but demonstrated photothermal activity at 808 nm^60, 61^.

**Figure 8:**
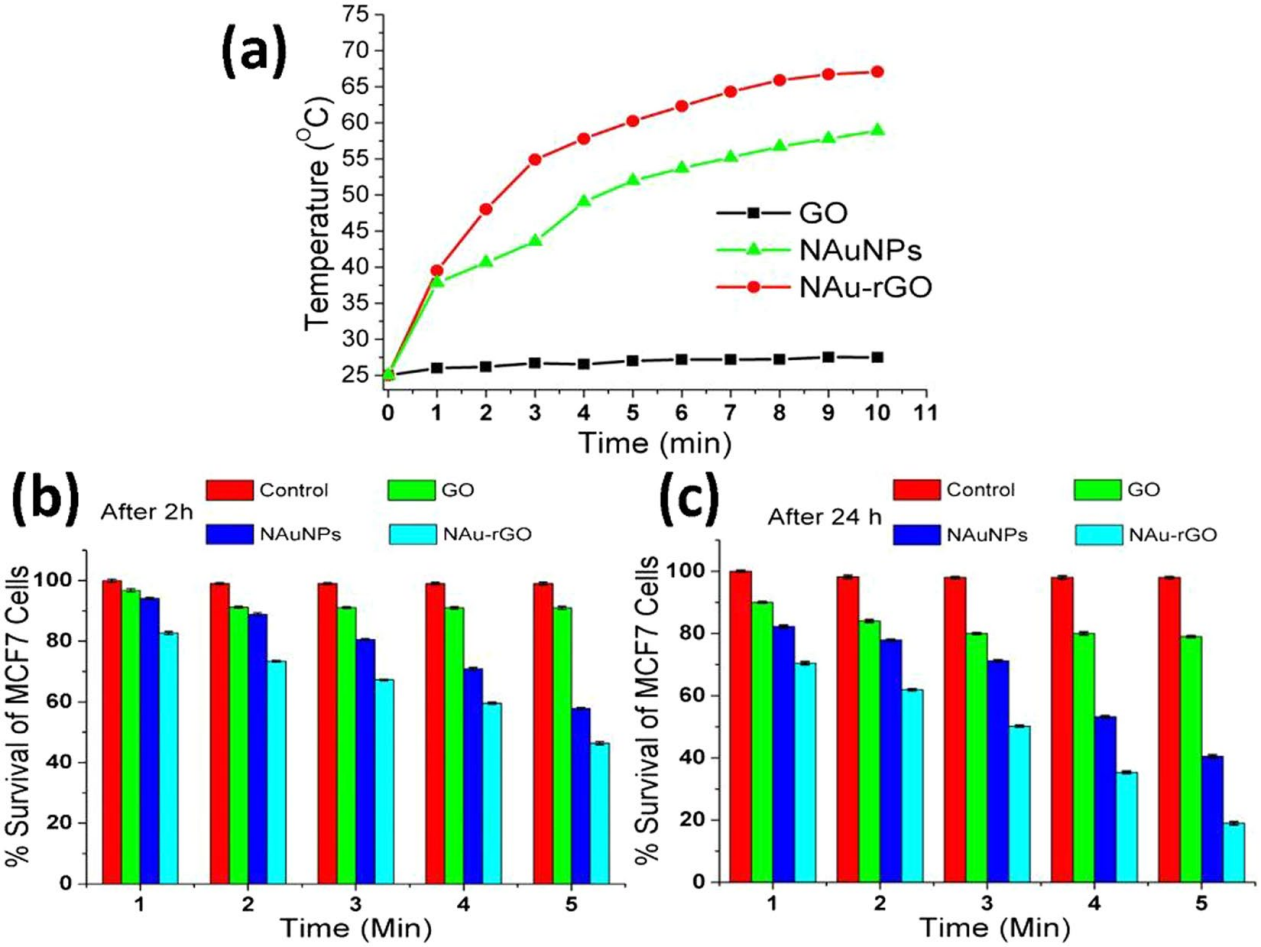
Photothermal response of the NAu-rGO nanocomposite to NIR exposure. (**a**) Temperature increase of medium containing GO, NAuNPs, and NAu-rGO nanocomposite. MTT assay for quantifying the percent survival of MCF7 breast cancer cells after photothermal therapy for 5 min with 10 μg/mL concentrations of GO, NAuNPs, and NAu-rGO nanocomposite for (b) 2 h and (c) 24 h post-treatment for different exposure times.

Further, the photothermal transduction efficacy of GO, NAuNPs, and NAu-rGO nanocomposite in cell medium was measured by irradiating the solution with a near infrared laser (laser power density of 0.5 W/cm^2^) containing GO, NAuNPs, and NAu-rGO nanocomposite (10 μg/mL) (Fig. 8a). The rapid rise in the temperature of the medium to 49 °C was observed within 2 min of laser exposure, while GO and NAuNPs were only at 25 °C and 38 °C, respectively, under the same irradiation conditions. Thus, GO showed much lower energy conversion efficiency after laser irradiation. After decoration of the NAuNPs on the GO sheets, spherical NAuNPs functioned as catalysts to improve the photothermal properties of GO. The photothermal conversion efficacy of NAuNPs, which showed a lower temperature increase compared to NAu-rGO nanocomposites, was also improved. The cumulative effect of the absorbance of NAu NPs and rGO in the NIR region (Supplementary Fig. 2) may be responsible for the photothermal activity of NAu-rGO nanocomposites. The Au NPs have a tendency to attach at defects and vacancies of rGO in NAu-rGO nanocomposites, which seizes the mobility of NAu NPs upon laser irradiation in the nanocomposite and consequently, there is no shape transformation of the spherical NAu NPs. Moreover, laser irradiation caused deoxygenation of rGO, which in turn increased the thermal conductivity and heat transfer capability of the rGO, which indeed improved the photothermal convergence properties of the NAu-rGO nanocomposites at 808 nm^62^.

Next, the photothermal conversion efficiency of NAu-rGO the nanocomposite was analyzed by MTT assay, where irradiation experiments were performed in presence of breast cancer cells (MCF7 cell line) and compared with GO and NAuNPs. Here, MCF7 cells were incubated with 10 μg/mL GO, NAuNPs, and NAu-rGO nanocomposite for effective interaction of the particles with the cells and then the medium was irradiated for different times (1–5 min). After exposure followed by incubation for 2 h, cells survival was analyzed by MTT assay (Fig. 8b). MCF7 cells showed only 50% survival after 2 h of incubation when irradiated for 5 min with 10 μg/mL NAu-rGO nanocomposite. In contrast, more than 90% survival of MCF7 cells was observed in the presence GO under the same reaction conditions. Further, these cells were incubated for 24 h and the percent survival of the cells was calculated. Less than 20% survival was observed after 24 h incubation in NAu-rGO nanocomposite-treated cells (Fig. 8c). In contrast, more than 80% and 45% survival rates of MCF7 cells were observed in the GO and NAuNPs, respectively, under the same reaction conditions as used for the NAu-rGO nanocomposite. Therefore, compared to GO and NAuNPs, the NAu-rGO nanocomposite showed improved and effective photothermal conversion efficacy and can be used for biomedical applications.

**Figure 9:**
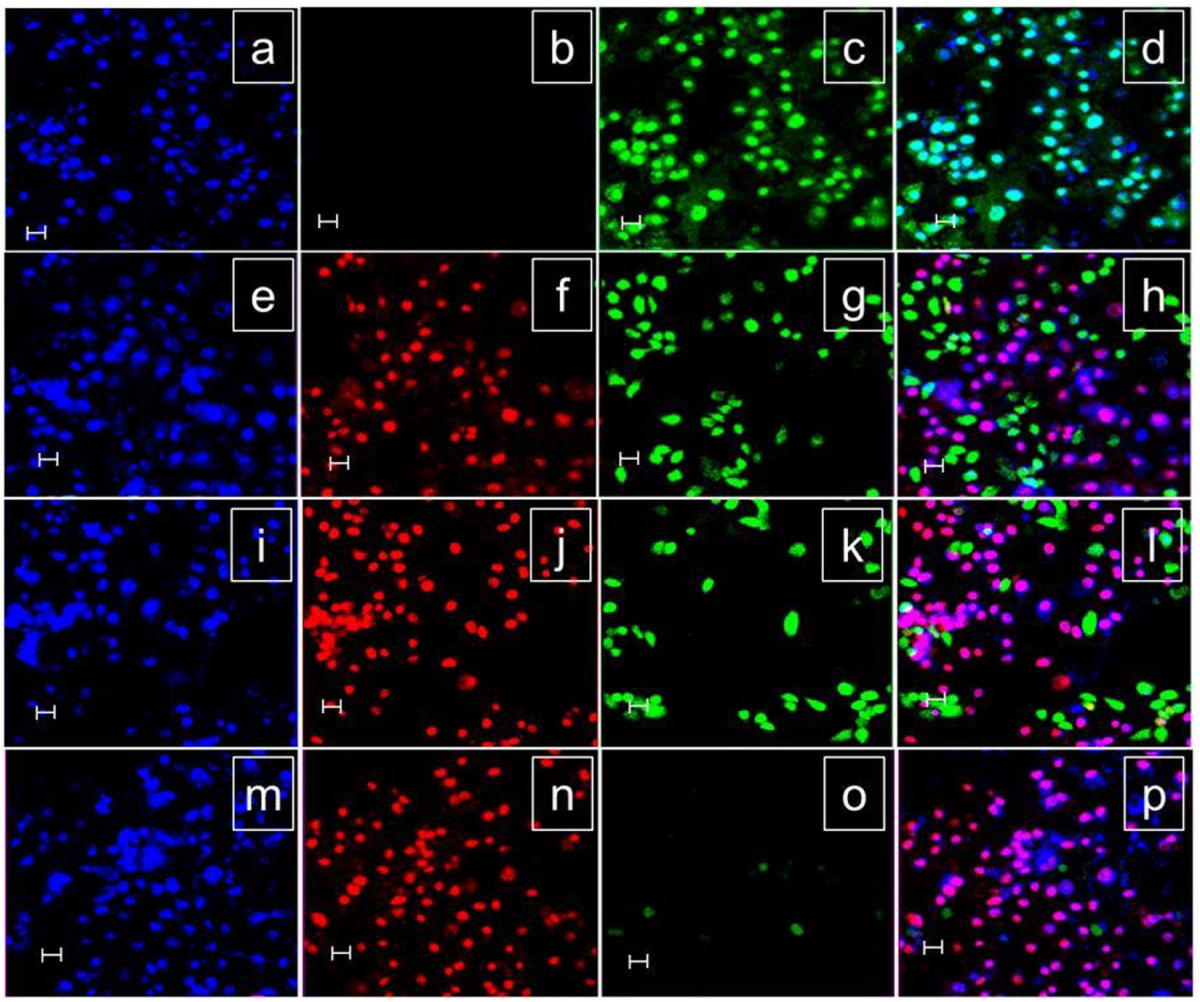
Confocal microscopy images of MCF7 cells. (**a–d**) in absence of GO and NAuNPs, NAu-rGO nanocomposite in presence of (**e–h**) GO, (**i–l**) NAuNPs, and (**m–p**) NAu-rGO nanocomposite, which were irradiated with an 800-nm laser with a power density of 0.5 W cm^2^ for 5 min and stained with DAPI, PI, and FDA after 24 h incubation (scale: 20 μM).

To demonstrate the therapeutic efficiency of the NAu-rGO nanocomposite, a qualitative live/dead cell assay was performed using PI, DAPI, and FDA dyes following irradiation of MCF7 cells with an 800-nm diode laser for 5 min (Fig. 9). Cells were observed by CLSM. The living cells cannot uptake the PI dye (nucleus penetrating) because of its intact cell membranes. In contrast, necrotic or early apoptotic cells can take up the PI. Therefore, for this study we used FDA dye to stain live cell nucleus, PI for dead cells and DAPI for nucleus of live and dead cells. MCF7 cells in absence of GO, NAuNPs, and NAu-rGO nanocomposite showed intact morphology as demonstrated by the blue and bright green fluorescence and absence of red fluorescence (Fig. 9a–d) after 24 h of incubation. Very few cells were affected by the photothermal activity of GO, as very few cells showed red fluorescence and a large number of cells showed green fluorescence (Fig. 9e–h). From the MTT assay results, NAuNPs clearly caused the death of more than 50% of MCF7 cells. This effect is shown in Fig. 9i–l, which shows an increase in the number of red fluorescent cells and lower number of green fluorescent cells. Although NAuNPs showed good photothermal activity against MCF7, the NAu-rGO nanocomposite exhibited remarkable photothermal transduction efficacy, as there was nearly no green fluorescence in CLSM analysis for MCF7 cells (Fig. 9m–p). Both the MTT assay and CLSM analysis showed that the as-prepared NAu-rGO nanocomposite have high potential as near infrared PTT agents and low cytotoxicity towards mammalian cells, and thus can be used for various biomedical applications such as antimicrobial agents and drug delivery vehicles.

## Conclusion

In summary, we developed a novel, rapid synthesis method for eco-friendly NAuNPs decorated with rGO nanocomposite using thermostable antimicrobial nisin peptide under autoclave conditions. Various analytical methods were used to confirm the reduction of Au^3+^ ions and GO. The as-prepared NAu-rGO nanocomposite showed charge transfer characteristics and photothermal energy conversion. Solution process sol-gel derive ZnO TFT memory devices with SiO_2_/NAu-rGO nanocomposite dielectric stack were developed. The transfer characteristics of the memory device showed a hysteresis window in dual sweep mode. The charge trapping and de-trapping on SiO_2_/NAu-rGO nanocomposite dielectric stack was verified by the shifts in threshold voltage by applying positive and negative bias voltage. *In vitro* cytotoxicity effects (MTT assay) of the NAu-rGO nanocomposite on the L929 and HeLa cell lines showed very low dose-dependent cytotoxic effects towards mammalian cell lines. The as-prepared nanocomposite showed very effective near infrared PTT activity compared to NAuNPs and rGO following 5-min exposure against MCF7 breast cancer cells, in which more than 80% cell growth was inhibited. The large-scale green production of the rGO and NAu-rGO nanocomposite using nisin thermostable peptides is more effective than existing chemical reduction methods and avoids the use of hazardous chemicals and harsh preparation methods.

## Methods

### Chemical reagents

All reagents were purchased from Sigma Aldrich Co. Ltd. (St. Louis, MO, USA) and were of analytical grade. HiMedia Laboratories Ltd. (Mumbai, Maharashtra, India) provided media components. The solubility of all chemicals in water was 100% and no further purification was required for experiments.

### Preparation of GO sheets

The method described by Hummers and Offeman was used to prepare GO by oxidizing natural graphite^63^. The aqueous brown solution of GO (4 mg/mL) was used for further experiments. For each experiment, GO sonication was performed to obtain homogeneous graphene sheets.

### Preparation of rGO and NAu-rGO nanocomposite

The NAu-rGO nanocomposite was synthesized by using thermostable nisin peptides (2.5% [w/w], approximately 1 × 10^6^ IU/g). In phosphate buffer (pH 6.8), 5 mg/mL of nisin peptides were added to an aqueous solution of 0.5 mM HAuCl_4_∙4H_2_O and incubated for 2 h at 25 °C room temperature. GO (0.8 mg/mL) was then dispersed in the above mixture under sonication for 1 h. The same method without HAuCl_4_ was employed to synthesize rGO. Both solutions were autoclaved at 121 °C under 15 lbs pressure for 15 min. The synthesis of rGO and NAu-rGO was monitored by UV-Vis spectroscopy.

### TFT-based non-volatile memory device

Zinc acetylacetonate hydrate (Sigma Aldrich) was dissolved in 2-methoxyethanol to produce a 0.1 M solution. The as-prepared precursors were stirred for 2 h to ensure that they were completely dissolved. For device fabrication, a B-doped p-type Si wafer (resistivity between 0.001 and 0.003 Ω-cm) served as the gate electrode, and 300 nm of thermally grown SiO_2_ was used as the dielectric layer. Prior to spin-coating, SiO_2_/Si substrates were cleaned ultrasonically in acetone and methanol for 10 min each. They were then rinsed with deionized water for 5 min. The previously prepared AuNPs-rGO nanocomposite solution was spin-coated onto the SiO_2_/Si substrates at 2000 rpm for 20 s. Next, ZnO solution was spin coated at 4000 rpm for 40 s to produce layers that were ~25 nm thick. The films were dried on a hot plate at 200 °C for 10 min to evaporate the solvents. Finally, the films were annealed for 1 h in a tube furnace at 400 °C to decompose and oxidize the precursors.

Finally, Al electrodes were thermally grown on the active layer using a shadow mask, with the channel width and length maintained at 1000 and 100 µm, respectively. The resulting source and drain electrodes were approximately 100 nm thick. Current–voltage (I–V) measurements were performed using a semiconductor analyser.

### Characterizations

UV-Vis spectra were measured with a UV 1800 spectrophotometer (Shimadzu, Kyoto, Japan). X-ray diffraction (XRD) studies of the nanocomposites were performed with an X-ray diffractometer (Bruker, Billerica, MA, USA) with Cu K_α_ radiation (λ-0.15418 nm). Raman spectra were collected from a Renishaw confocal microscope Raman system with laser 532 nm (Gloucestershire, UK). Field-emission scanning electron microscope images were obtained with a Carl Zeiss 200 FEG FESEM (Jena, Germany). Transmission electron microscopy (TEM) and high-resolution TEM (HRTEM) analyses were performed using a JEM-3010 (JEOL, Tokyo, Japan) operated at an accelerating voltage of 300 kV. X-ray photoelectron spectroscopy (XPS) analysis was performed on a PHI 5000 Versa Probe (ULVAC PHI, Chigasaki, Japan) spectrometer using Al K_α_ as the irradiation source. Current–voltage (I–V) measurements were performed using a semiconductor analyzer (Keithley 4200, Tektronix, Beaverton, OR, USA) with a probe station.

### Cell Culture

Human cervical cancer cells (HeLa) and human breast adenocarcinoma cells (MCF7) were purchased from ATCC (Manassas, VA, USA), and sub-cultured in McCoy’s 5 A medium containing fetal bovine serum (10%) and antibiotics (100 g/mL penicillin and 100 μg/mL streptomycin) from Sigma (St. Louis, MO, USA). In 25-cm^2^ tissue culture flasks, the cells were enriched and kept in an incubator at 37 °C with CO_2_ (5%) humidified atmosphere. After 90% confluence of the cells, Dulbecco’s phosphate-buffered saline (DPBS) was used to wash these cells. To detach the cells, 2 mL of trypsin−EDTA solution (0.25%) was applied and finally these cells were dispersed in complete medium (10 mL).

### Toxicity analysis

The percent viability of the cells was quantified by MTT assay where the cells (2 × 10^5^ cells/mL) were incubated with 3-(4,5-dimethylthiazol-2-yl)-2,5-diphenyltetrazolium bromide (MTT) solution and colorimetrically observed for the mitochondrial oxidation of MTT using EZ-CYTOX-Enhanced Cell Viability Assay Kit. The cells were incubated in DMEM (Dulbecco’s modified Eagle Medium) with different concentrations of NAu-rGO nanocomposite (10, 20, 40, 60, 80 and 100 μg/mL). After incubation for 24 and 48 h, NAu-rGO nanocomposite with medium was removed and freshly prepared 10 μL MTT solution was added. The cells were further incubated for 4 h and then treated with dimethyl sulfoxide (100 μL). Finally, the absorbance at 405 nm was measured for each well using an ELISA plate reader (BIORAD, USA).

### *In-vitro* photothermal therapy

*In-vitro* photothermal conversion efficacy was analysed with an 800-nm infrared diode laser with a power density of 0.5 W/cm^2^ (Changchun New Industries Optoelectronics Technology Co., Ltd., Changchun, China) and a thermometer (UNI-T 1310, UNI-T Electronic Corp., Dongguan, China). MCF7 breast cancer cells incubated with rGO, NAuNP, and NAu-rGO nanocomposites for 24 h were exposed to an 800-nm diode laser (power = 0.5 W/cm^2^) for 1–5 min and allowed to grow for 24 h. Following laser illumination, the cells were incubated with the fluorescent labels 4′,6-diamidino-2-phenylindole (DAPI), propidium iodide (PI), and fluorescein diacetate (FDA) dyes to produce blue, red, and green fluorescence from live and dead cells, respectively, for visualization under a confocal laser scanning microscope (CLSM). Cell viability was quantified using the MTT assay at 2 and 24 h post-treatment with the laser.

## Electronic supplementary material

Supplementary information
