# Supplementary material for: Rapid synthesis and decoration of reduced graphene oxide with gold nanoparticles by thermostable peptides for memory device and photothermal applications
Source: Sci Rep. 2017 Sep 8;7:10980. doi: 10.1038/s41598-017-10777-1 (PMC5591228; doi:10.1038/s41598-017-10777-1)
Supplement: Supplementary file 1 — Supplementary information [file 41598_2017_10777_MOESM1_ESM.doc]

**Rapid synthesis and decoration of reduced graphene oxide with gold nanoparticles by thermostable peptides for memory device and photothermal applications**

Sachin V. Otari^a,b^, Manoj Kumar^c^, Muhammad Zahid Anwar^b^, Nanasaheb D. Thorat^c^, Sanjay K. S. Patel^b^, Dongjin Lee^c^, Jai Hyo Lee^c^, Jung-Kul Lee^b*^, Yun Chan Kang^e*^, Liaoyuan Zhang^a,b*^

^a^ Key Laboratory of Biopesticide and Chemical Biology, Fujian Agriculture and Forestry University, Fuzhou, Fujian province, 350002, PR China

^b^ Department of Chemical Engineering, Konkuk University, Seoul 05029, Republic of Korea

^c^ Department of Mechanical Engineering, Konkuk University, Seoul 05029, Republic of Korea

^d^ Materials & Surface Science, Institute Bernal Institute, University of Limerick, Limerick, Ireland

^e^ Department of Materials Science and Engineering, Korea University, Seoul 02841, Republic of Korea

*Corresponding author:

Mailing address: Department of Mechanical Engineering, Konkuk University, Seoul 05029, Republic of Korea. Tel: 82-2-450-3505. Fax: 82-2-458-3504. E-mail: jkrhee@konkuk.ac.kr

Department of Materials Science and Engineering, Korea University, Seoul 02841, Republic of Korea. Tel: 82-2-3290-3268. Fax: 82-2-928-3584. E-mail: yckang@korea.ac.kr

Key Laboratory of Biopesticide and Chemical Biology, Fujian Agriculture and Forestry University, Fuzhou 350002, PR China. Tel: 13-6-0086-4845. Fax: 13-6-0086-4832. E-mail: zliaoyuan@126.com

**Figures:**


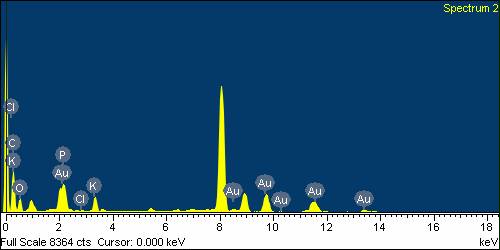


**Supplementary Figure 1:** **EDS spectra of NAu-rGO nanocomposite** confirming formed spherical nanoparticles are of NAu NPs.


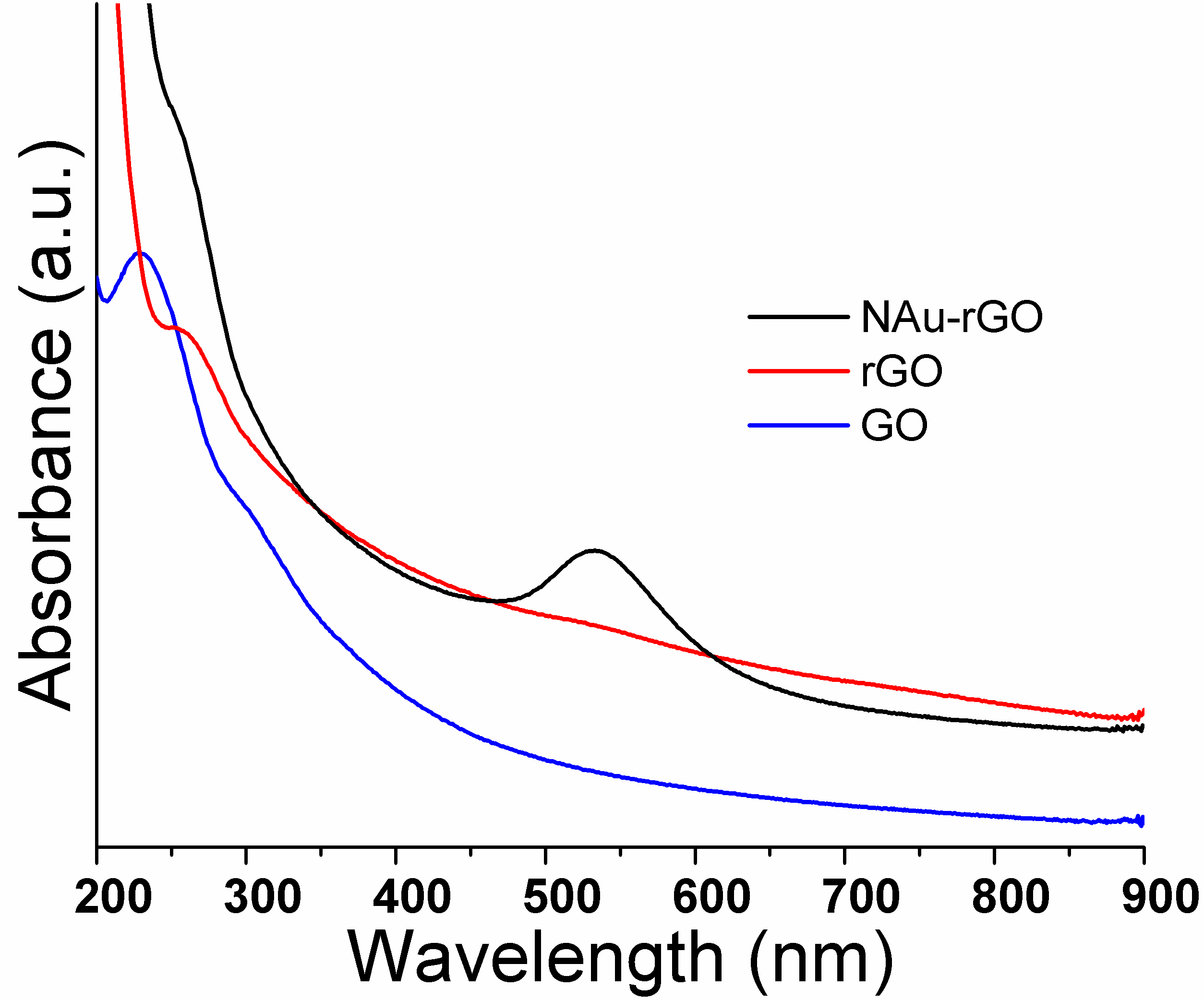


**Supplementary Figure 2:** **Absorption spectra of GO, rGO and NAu-rGO nanocomposite from UV to NIR wavelength.** The rGO and NAu-rGO nanocomposite are having no absorbance in NIR region and NAu-rGO nanocomposite showed absorbance at ~540 nm.
